# Calorimetric Thermoelectric Gas Sensor for the Detection of Hydrogen, Methane and Mixed Gases

**DOI:** 10.3390/s140508350

**Published:** 2014-05-09

**Authors:** Nam-Hee Park, Takafumi Akamatsu, Toshio Itoh, Noriya Izu, Woosuck Shin

**Affiliations:** National Institute of Advanced Industrial Science and Technology (AIST), Shimo-Shidami, Moriyama-ku, Nagoya 4638560, Japan; E-Mails: namhee.park@aist.go.jp (N.-H.P.); t-akamatsu@aist.go.jp (T.A.); itoh-toshio@aist.go.jp (T.I.); n-izu@aist.go.jp (N.I.)

**Keywords:** calorimeter, thermoelectric gas sensor, dual catalyst, methane, hydrogen

## Abstract

A novel miniaturized calorimeter-type sensor device with a dual-catalyst structure was fabricated by integrating different catalysts on the hot (Pd/*θ*-Al_2_O_3_) and cold (Pt/*α*-Al_2_O_3_) ends of the device. The device comprises a calorimeter with a thermoelectric gas sensor (calorimetric-TGS), combining catalytic combustion and thermoelectric technologies. Its response for a model fuel gas of hydrogen and methane was investigated with various combustor catalyst compositions. The calorimetric-TGS devices detected H_2_, CH_4_, and a mixture of the two with concentrations ranging between 200 and 2000 ppm at temperatures of 100–400 °C, in terms of the calorie content of the gases. It was necessary to reduce the much higher response voltage of the TGS to H_2_ compared to CH_4_. We enhanced the H_2_ combustion on the cold side so that the temperature differences and response voltages to H_2_ were reduced. The device response to H_2_ combustion was reduced by 50% by controlling the Pt concentration in the Pt/*α*-Al_2_O_3_ catalyst on the cold side to 3 wt%.

## 1. Introduction

Gas calorimeters, which measure the calorific values or Wobbe Index (WI) of gases [1], are used to control the thermal input in various types of steel mill and petrochemical industry furnaces, as well as control the fuel in power plant turbines. Conventional calorimeters for fuel gas, natural gas, or coal gasification products are highly complex, sophisticated apparatus for monitoring the heats of combustion of fuels in the burners or turbines of power plants [2]. Typical calorimeters are large, self-standing units that are used to monitor large-scale systems, and there are no smaller calorimeters appropriate for the various burners or turbines of smaller-scale systems.

A new sensor device is presented in this study which contains all the functional elements of a conventional thermocouple-type calorimeter (temperature sensor, catalytic combustor, and heater). The device, essentially a calorimeter of a thermoelectric gas sensor (calorimetric-TGS), combines catalytic combustion and thermoelectric technologies [3]. The sensor is suitable for low-cost production and advantageous in terms of short start-up and response times [4]. Detailed features of thermoelectric sensor technologies have been described previously [5].

Natural gas consists mostly of methane, along with lesser amounts of ethane and propane. Noncombustible components such as carbon dioxide and nitrogen may also be present. Several reports have described the typical compositions of natural gas [6]. In the case of coal gas, the principal component, CO, is reacted with H_2_O and produced to H_2_ and CO_2_ before the gas flows into the burner or turbine [7].

Considering fuel gas compositions, TGS sensors must be able to properly combust H_2_, CO and CH_4_. The detection of H_2_ and CO by TGS has been demonstrated previously [3,8], but the detection of CH_4_ is challenging as it requires a higher catalyst temperature [9]. At lower catalyst temperatures, the heat of combustion catalyst for H_2_ exceeds that of CH_4_, causing the ratio of combustion heat to sensor output to deviate significantly from linearity.

In the present study, we integrated modified catalytic combustors for CH_4_ to balance the combustion calories-to-sensor outputs for more reliable calorimetric applications. The fabrication, characterization, and gas responses of the micro calorimetric-TGS devices, which utilize a thermoelectric thin film, were investigated with various catalytic combustor material sets and operating temperatures. The responses of the TGS to the combustion heat for different compositions of gases were also investigated.

## 2. Experimental Section

### 2.1. Structure and Working Principle of the Dual-Catalyst Calorimetric-TGS Devices

**Figure 1.**
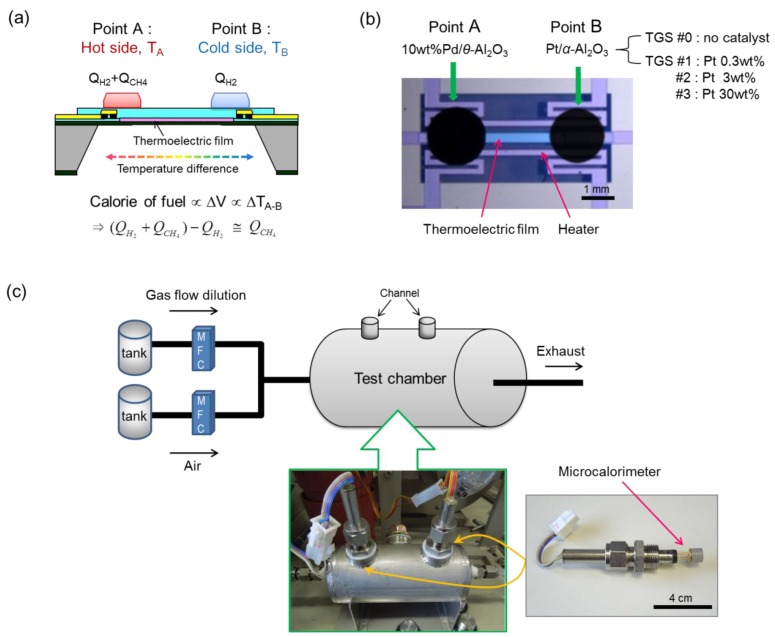
(**a**) Schematic illustration of device structure and working principle, and (**b**) photograph of a calorimetric-TGS device. (**c**) Schematic and photograph of the measurement system for the calorimetric-TGS devices.

Our purpose was to design and demonstrate a calorimetric-TGS with a dual-catalyst structure to balance the combustion calories for H_2_ and CH_4_ in a gas fuel. To this end, Pd/*θ*-Al_2_O_3_ and Pt/*α*-Al_2_O_3_ were deposited on the hot (point A) and cold (point B) sides of a membrane, respectively, as depicted in Figure 1a. The Pd/*θ*-Al_2_O_3_ catalyst oxidizes both H_2_ and CH_4_ gases well, and the Pt/*α*-Al_2_O_3_ catalyst is the most well-known catalytic combustor for hydrogen, with high activity toward its selective oxidation. The role of the Pt/*α*-Al_2_O_3_ catalyst at point B is to improve the heat of H_2_ catalytic combustor (*Q*_*H*_2__) on the cold side and to reduce the temperature difference between the hot and cold sides. The structure and working principle of the dual-catalyst calorimetric-TGS are based on our previous study of a micro-thermoelectric hydrogen sensor (micro-THS) with a dual catalyst for H_2_ and CO [10]. When a mixture of H_2_ and CH_4_ is introduced into the calorimetric-TGS device, the Pd/*θ*-Al_2_O_3_ catalyst releases the heats of combustion for H_2_ and CH_4_ (*Q*_*H*_2__+ *Q*_*CH*_4__) on the hot side (point A), and the heat of combustion of H_2_ (*Q*_*H*_2__) is generated by combustion on the Pt/*α*-Al_2_O_3_ catalyst on the cold side (point B). A temperature difference is developed between points A and B, which is then converted into a voltage by the Seebeck effect across the thermoelectric film. Therefore, the combustion heat (*Q*_*CH*_4__) can be obtained by subtracting the combustion heat for H_2_ on the Pt/*α*-Al_2_O_3_ catalyst at point B (*Q*_*H*_2__) from the total combustion heat for both H_2_ and CH_4_ on the Pd/*θ*-Al_2_O_3_ catalyst (*Q*_*H*_2__ + *Q*_*CH*_4__) at point A.

### 2.2. Preparation of Calorimetric-TGS Devices with Various Catalytic Combustors

The size of the calorimetric-TGS chip was 4 × 4 mm^2^, and a double-sided polished Si wafer with 0.35 mm thickness was used as substrate. A silicon-germanium (SiGe) thin film [11] was deposited by DC magnetron sputtering and patterned into the thermoelectric (TE) element by RIE etching. The membrane was designed to be 1.2 × 0.8 mm^2^. The membrane and catalytic combustors of the calorimetric-TGS were fabricated based on our previous studies [5]. The combustion catalysts for the devices were prepared by the colloidal method. For the Pd/*θ*-Al_2_O_3_ catalyst powder, commercially available *θ*-Al_2_O_3_ powder (Taimei Chemicals Co., Ltd., Nagano, Japan; average particle size: 14 nm) was added into 4 wt% Pd-PVP colloids (Pd colloids stabilized in poly(*N*-vinyl-2-pyrrolidone (PVP); Tanaka Precious Metals Co., Ltd., Tokyo, Japan; average Pd nanoparticle size: 4 nm) with mechanical stirring. Analogously, the Pt/*α*-Al_2_O_3_ catalyst powder was prepared by mixing commercially available *α*-Al_2_O_3_ powder (Taimei; avg. particle size: 100 nm) with 4 wt% Pt-PVP colloid (Pt colloids stabilized in PVP; Tanaka; avg. Pt nanoparticle size: 2 nm). The Pd concentration was fixed at 10 wt% and the Pt concentration was varied from 0.3 to 30 wt%. Each mixture was stirred at 80 °C for 1 h. The obtained catalyst pastes were dried in an oven at 90 °C for 30 min and subsequently heated at 300 °C for 2 h in air. The catalysts powders were mixed with an organic vehicle, *i.e.*, a 9:1 w/w mixture of terpineol and ethyl cellulose, to prepare the dispensing pastes. The pastes were deposited on points A and B of the calorimetric-TGS devices by a dispenser system (Musashi Engineering, Inc., Aichi, Japan), as shown in the photograph in Figure 1b. The thicknesses of the Pd/*θ*-Al_2_O_3_ and Pt/*α*-Al_2_O_3_ catalysts were controlled to be about 25 and 3 μm, respectively. The deposited catalyst pastes were heated at 300 °C for 2 h in air. The compositions and names of the prepared devices with the various catalytic combustors are given in Table 1.

### 2.3. Characterization, Gas Response Testing and Combustion Heat

The catalyst surface temperatures on the calorimetric-TGS devices were monitored by IR camera (LAIRD-270A, Nikon, Tokyo, Japan) and the power consumption was estimated from surface temperature. The emissivity of the catalyst was considered and calibrated before the measurement. The combustion performance of the calorimetric-TGS devices was investigated in a gas-flow type test chamber, as shown in Figure 1c. The voltage signals from the devices were recorded with a digital multimeter/data acquisition system (Model 2700, Keithley Instruments, Cleveland, OH, USA) by flowing CH_4_, H_2_, and a CH_4_ + H_2_ gas mixture at a rate of 200 mL/min in the test chamber (2% CH_4_/N_2_ and 2% H_2_/N_2_ standard gases; DAIWA Shokai Co., Ltd., Osak, Japan). The voltage signal, Δ*V*, is considered as an index of the combustion performance of the catalyst in the calorimetric-TGS device, and is evaluated as follows:

(1) ΔV=α⋅ΔT

where Δ*V* is the voltage signal, *α* is Seebeck coefficient of the thermoelectric film, and Δ*T* is the temperature difference between points A and B, as shown in the Figure 1a and b. The response voltage (Δ*V*) of a calorimetric-TGS device for gas combustion is defined as the difference between the voltage output of the inflammable gas in air (*V_gas_*) and the offset voltage in air (*V_air_*), which is given by the follow formula:

(2) $$\Delta V=V_{\text{gas}}-V_{\text{air}}$$

Finally, the combustion heats of the gas fuels were calculated for the calorimetric-TGS devices.

## 3. Results and Discussion

### 3.1. Power Consumption

**Figure 2.**
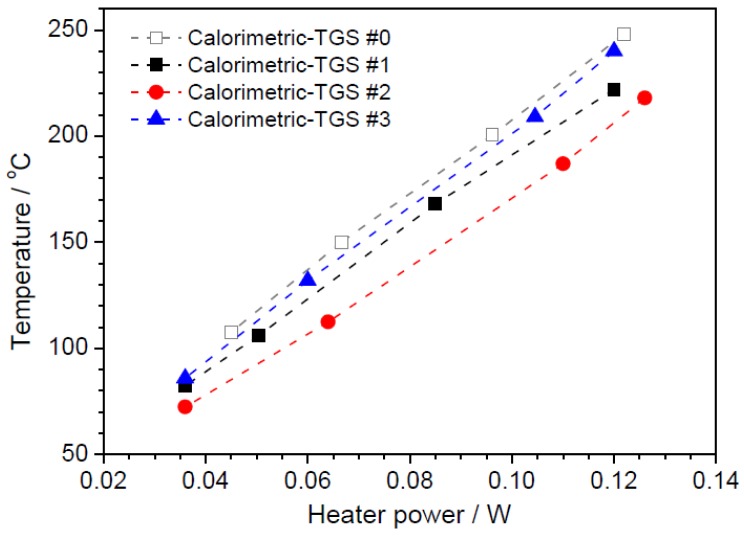
Catalyst temperature as a function of power consumption, as estimated from the integrated catalyst surface by IR camera monitoring.

The surface temperatures of the Pd/*θ*-Al_2_O_3_ catalysts in the calorimetric-TGS devices were determined by monitoring with the IR camera. Figure 2 shows the catalyst temperatures in the range 50–250 °C estimated from the surface temperatures as a function of the power consumption of the devices. For devices #0, #1, #2 and #3, the power consumptions display good linearity, requiring 0.122, 0.12, 0.126 and 0.12 W to heat the catalysts to 248, 222, 218 and 240 °C, respectively.

### 3.2. Combustion Performance with H_2_, CH_4_ and the Mixed Gases

**Figure 3.**
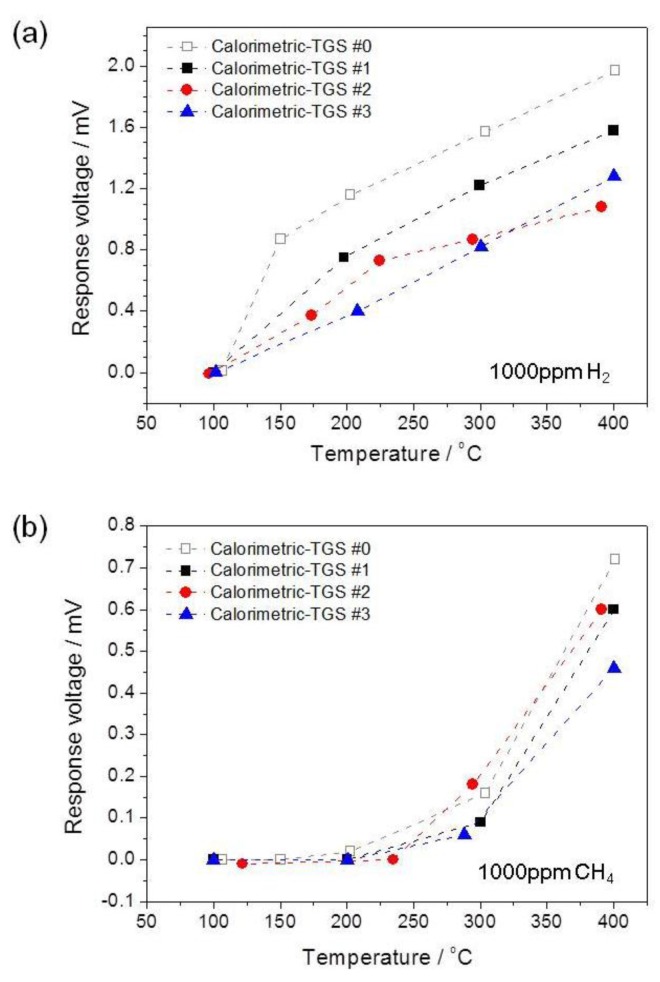
Temperature dependence of the response voltages Δ*V* over the temperature range 100–400 °C for (**a**) 1000 ppm H_2_ and (**b**) 1000 ppm CH_4_ in air.

Figure 3 shows the temperature dependence of the response voltages Δ*V* of the devices in the temperature range 100–400 °C for flows of (a) 1000 ppm H_2_ and (b) 1000 ppm CH_4_ in air. The calorimetric-TGS devices display linear relationships between the temperature increases and the response voltages to H_2_, except for the device #2. As the Pt concentration in the Pt/*α*-Al_2_O_3_ catalyst at point B increases, the Δ*V* for H_2_ of the device decreases in the low temperature range below 250 °C, as shown in Figure 3a. This result indicates that the H_2_ combustion performance of the Pt/*α*-Al_2_O_3_ catalyst improves by increasing the Pt concentration. The temperature differences between the Pd and Pt catalysts decrease due to the active combustion by the Pt/*α*-Al_2_O_3_ catalysts with higher Pt concentrations at point B, consequently, Δ*V* of devices decreases. However, at temperatures over 300 °C, the combustion performance on point B of device #2, containing 3 wt% Pt/*α*-Al_2_O_3_, becomes lower than that of the other devices. The response voltage of the calorimetric-TGS #2 at 291 °C is close to that of calorimetric-TGS #3 with 30 wt% Pt/*α*-Al_2_O_3_ at 300 °C. Device #2 subsequently shows the lowest Δ*V* of 1.08 mV at 391 °C, indicating the high combustion of H_2_ on the 3 wt% Pt/*α*-Al_2_O_3_ catalyst. At 400 °C, the response voltages of devices #0, #1 and #3 are 1.97, 1.58 and 1.28 mV, respectively. It indicates that the catalytic activity and H_2_ selectivity of the 3 wt% Pt/*α*-Al_2_O_3_ catalyst is higher than those of the 30 wt% Pt/*α*-Al_2_O_3_ catalyst at B point, and the study for the catalytic performance of Pt/alumina catalysts with the various concentration of Pt confirms previously reported results [12].

In contrast, the catalysts of the calorimetric-TGS devices start to burn 1000 ppm CH_4_ at temperatures over 200 °C, as shown in Figure 3b. The Δ*V* for 1000 ppm CH_4_ for devices #0, #1, #2 and #3 are 0.16, 0.09, 0.18 and 0.06 mV at 303, 300, 294 and 288 °C, respectively. At temperatures over 300 °C, the calorimetric-TGS #1 shows a rapid increase in Δ*V* to 0.6 mV at 400 °C. The combustion performance of the 0.3 wt% Pt/*α*-Al_2_O_3_ catalyst at point B is significantly lower than that of the 10 wt% Pd/*θ*-Al_2_O_3_ catalyst at point A at high temperatures for calorimetric-TGS #1, compared to those of devices #2 and #3. However, device #3 shows the lowest response voltage to CH_4_ (0.46 mV) at 400 °C, indicating its high CH_4_ combustion performance. Furthermore, the 30 wt% Pt/*α*-Al_2_O_3_ catalyst at point B in device #3 has higher CH_4_ combustion performance than the 3 wt% catalyst in device #2. Device #0 is no longer tested in following sections and we will discuss only the combustion performance of the devices with dual catalyst, because it confirms the worst combustion performance to H_2_ and CH_4_ of device #0 compared to other calorimetric-TGS devices in Figure 3.

**Figure 4.**
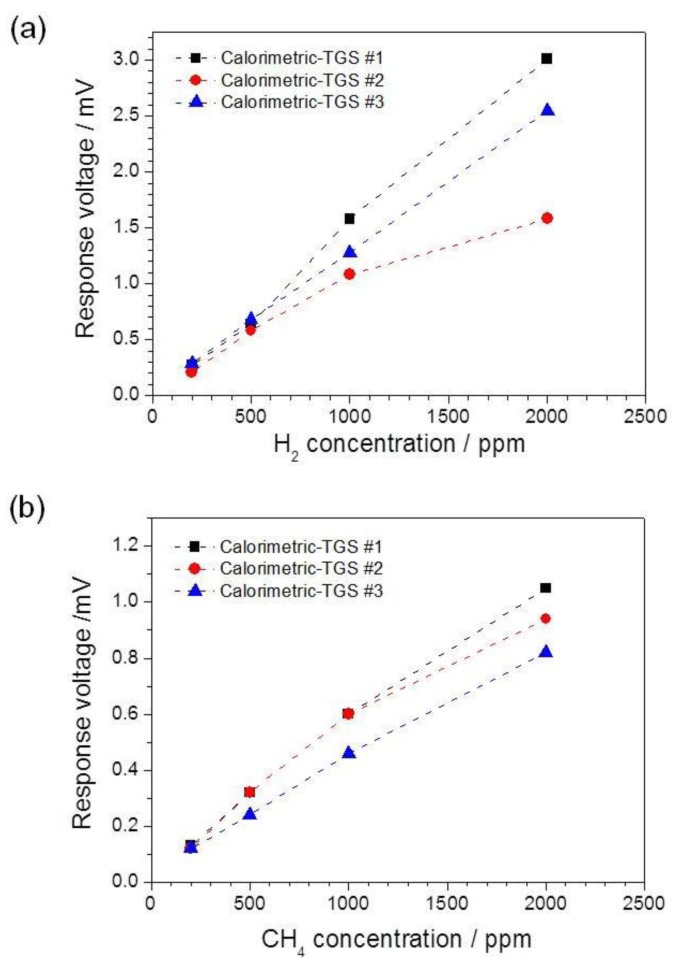
Response voltage Δ*V* as a function of gas concentration for (**a**) H_2_ and (**b**) CH_4_. The operating temperatures of the calorimetric-TGSs #1, #2 and #3 were 400, 391 and 400 °C, respectively.

The response voltages of the devices as a function of H_2_ and CH_4_ concentrations are shown in Figure 4. The operating temperatures for the calorimetric-TGSs #1, #2 and #3 were 400, 391 and 400 °C, respectively, and the introduced H_2_ and CH_4_ concentrations varied from 200 to 2000 ppm in air. The calorimetric-TGS devices show linear relationships between Δ*V* and the gas concentrations over the gas concentration ranges 200–1000 ppm for both H_2_ and CH_4_, as shown in Figure 4a and b, respectively. For H_2_ combustion performance *versus* gas concentration, the calorimetric-TGS #2 shows a lower Δ*V* than the other devices (Figure 4a). This means that the 3 wt% Pt/*α*-Al_2_O_3_ catalyst in device #2 burns H_2_ much better than the other Pt/*α*-Al_2_O_3_ catalyst compositions upon increasing gas concentration at 391 °C. However, the 3 wt% Pt/*α*-Al_2_O_3_ catalyst in device #2 did not exhibit a high CH_4_ combustion performance; in this case, the 30 wt% catalyst in device #3 performed better, giving the lowest Δ*V* among the devices (Figure 4b).

**Figure 5.**
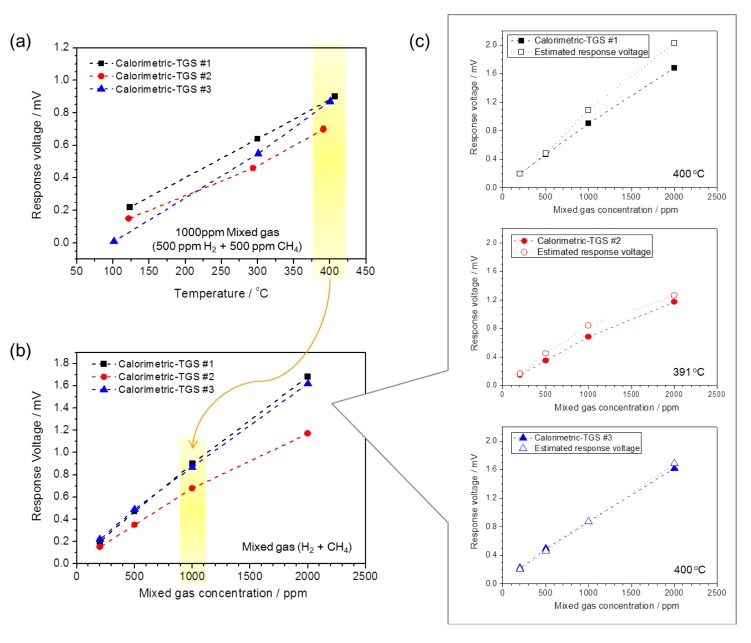
(**a**) Temperature dependence of the response voltage Δ*V* to 1000 ppm mixed gases (500 ppm CH_4_ + 500 ppm H_2_) in air over the temperature range 100–400 °C. (**b**) Δ*V* as a function of the mixed gas concentration at 400, 391 and 400 °C for devices #1, #2 and #3. (**c**) The estimated Δ*V* to the mixed gases, calculated from the experimental data for the response voltages to H_2_ and CH_4_ from Figure 4, using the formula: [(Δ*V* to H_2_) + (Δ*V* to CH_4_)]/2.

The temperature dependence of Δ*V* with 1000 ppm mixed gas (H_2_:CH_4_ = 50:50) for the three devices in the temperature range 100–400 °C is shown in Figure 5a. Here, Δ*V* increases linearly with temperature. The calorimetric-TGS #2 shows lower response voltages than devices #1 and #3: 0.46 mV at 294 °C and 0.7 mV at 391 °C. This indicates that the H_2_ combustion performance of the 3 wt% Pt/*α*-Al_2_O_3_ is much higher than that for CH_4_ in the mixed gas for the calorimetric-TGS #2, although the Pt/*α*-Al_2_O_3_ catalyst of device #3 has a higher Pt concentration than device #2. The combustion performance toward H_2_ at point B with the 3 wt% Pt/*α*-Al_2_O_3_ catalyst seems to be enhanced over the 30 wt% catalyst.

Figure 5b shows the Δ*V* to the mixed gases for the calorimetric-TGS devices as a function of gas concentration. The operating temperatures for the calorimetric-TGSs #1, #2 and #3 are 400, 391 and 400 °C, respectively, and the mixed gas concentration (H_2_:CH_4_ = 50:50) is varied from 200 to 2000 ppm in air. The Δ*V* is proportional to the mixed gas concentration for the calorimetric-TGS devices. These results are compared (Figure 5c) to the estimated response voltages, calculated using [(Δ*V* to H_2_) + (Δ*V* to CH_4_)]/2 with the experimental data from the response voltages to H_2_ and CH_4_. These findings confirm that the gas concentration-dependent response voltages to the mixed gases for the calorimetric-TGS devices correspond well to the estimated response voltages.

**Figure 6.**
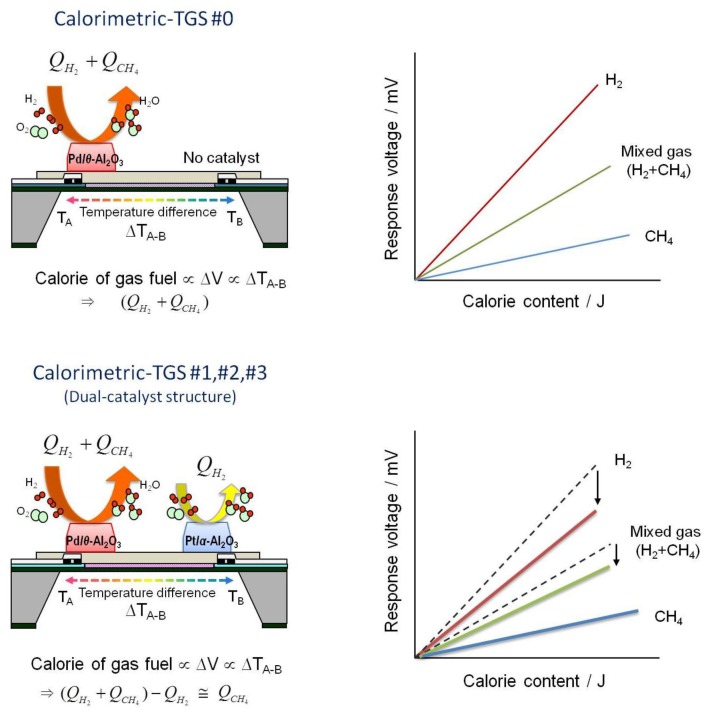
Schematic illustrations of the combustion mechanisms of the dual-catalyst calorimetric-TGS devices for mixed H_2_ and CH_4_ in air (top left and bottom left), and the expected graphs of gas calories-to-response voltages (top right and bottom right).

Figure 6 shows a schematic illustration of the combustion mechanism of the dual-catalyst calorimetric-TGS devices for mixed H_2_ and CH_4_ in air, and the expected graphs of gas calorie contents to response voltages. When the mixed gases flow, the calorimetric-TGS #0 device combusts both H_2_ and CH_4_, and a temperature difference is developed between points A and B due to the heats of combustion (*Q*_*H*_2__ + *Q*_*CH*_4__) of both H_2_ and CH_4_ at point A (top left, Figure 6). For this device, a graph of the expected gas calorie-to-response voltage is shown in the top right of Figure 6. On the other hand, in the dual-catalyst calorimetric-TGS devices #1–3, the temperature differences between points A and B can be reduced by lowering the excessive heats of combustion due to H_2_ (*Q*_*H*_2__), to balance with the heats of CH_4_ combustion (*Q*_*CH*_4__). Therefore, the gas calorie-to-response voltage graph can be expected as shown in the bottom right of Figure 6.

### 3.3. Responses to Combustion Calories by the Calorimetric-TGS Devices

**Figure 7.**
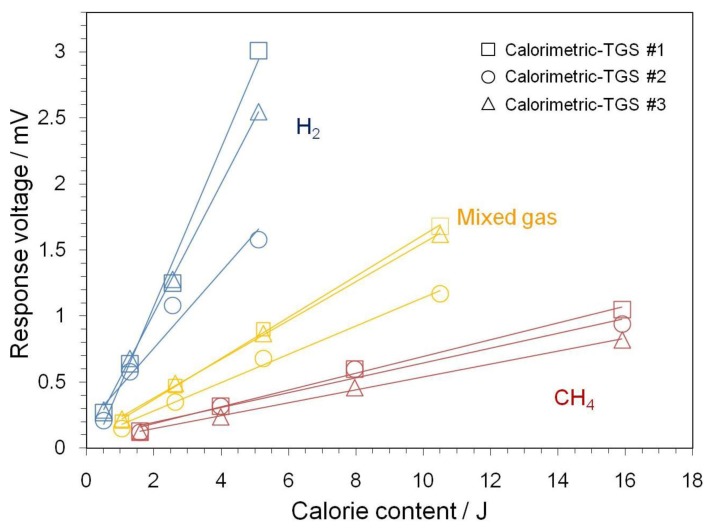
Combustion heats of calorimetric-TGS devices with various catalytic combustor compositions. The operating temperatures of devices #1, #2 and #3 are 400, 391 and 400 °C, respectively. Gas concentrations are 200, 500, 1000 and 2000 ppm for H_2_, CH_4_ and the gas mixture.

Graphs of the observed gas combustion calories to the response voltages toward H_2_, CH_4_, and the mixed gases are shown in Figure 7, which were plotted using results obtained at operating temperatures of 400, 391 and 400 °C for the calorimetric-TGSs #1, #2 and #3, respectively. The graphs show the gas detection performance of the calorimetric-TGS devices from 200 to 2000 ppm gas in air (from 0.51 J for 200 ppm H_2_ to 15.9 J for 2000 ppm CH_4_), confirming the very linear responses to the calorie content. The response to the combustion heat for H_2_ decreases with the Pt concentration in the Pt/*α*-Al_2_O_3_ catalyst. The response to CH_4_ combustion heat increases from 0.12 to 1.05 mV in the combustion calorie range of 1.59 and 15.9 J. To date, research is limited on the detection of CH_4_. Fleischer *et al.* [13] reported the detection of CH_4_ using a Ga_2_O_3_ thin film at high temperature (300–800 °C), which had a sensitivity of about 5 or 80 ppm to 5000 ppm CH_4_ in air at 420 and 740 °C, respectively. Sun *et al.* [14] reported that CH_4_ gas burns on SnO_2_ materials at temperatures above 600 °C, and Liu *et al.* [15] reported CH_4_ detection at 450 °C. However, although CH_4_ detection has been very difficult in the low temperature range (below 400 °C), our calorimetric-TGSs successfully detected CH_4_ at temperatures below 400°C. The responses to combustion heat, calorie content, for the mixed gases provide reasonable results with respect to the each gas (50% H_2_ and 50% CH_4_) in the mixture. Consideration of the thermal resolution of the calorimetric-TGS devices demonstrated that the sensor could detect 200 ppm H_2_ (0.51 J) in air; thus, we can conclude that the resolution is sufficiently high for calorimetric applications. High resolution performance in the device is predicted for the detection of the heats of dilute fuel gases or for very small samplings of fuel gases.

The calorimetric calibration of the calorimetric-TGS devices, which is an important part of their characterization, can be expressed by the following equation:

(3) Q∝ΔV

which can be rewritten as:

(4) Q=K⋅ΔV

where *Q*, *K*, and Δ*V* are the combustion heat, a response-to-combustion heat conversion factor, and the response voltage, respectively. Response-to-combustion heat conversion factors (*K*_H_2__, *K*_CH_4__, and *K_Mixedgas_*) are calculated from the measurement of each gas (H_2_, CH_4_, and mixed gases), and the results are shown in Table 2. The calibration functions of the calorimetric-TGS devices are linear between gas concentrations of 200 to 2000 ppm for each gas as shown in the table. Although the calorimetric-TGS devices show various response-to-combustion heat conversion factors for H_2_, CH_4_, and the mixed gases, device #2 shows relatively smaller differences in its conversion factors for all three gas flows than the other devices.

Ideally, a calorimeter should have a constant response-to-combustion heat conversion factor for each specific gas in a fuel, showing for each data point its response to the combustion heats of the gases plotted on the same line. This study is a step toward an ideal calorimeter for reliable application. These calorimetric-TGS devices have successfully demonstrated that the H_2_ combustion heat, which is easily generated at low temperature, can be reduced by up to 50% and the total observed response approaches that of CH_4_ more closely. It is worth noting that the dual-catalyst calorimetric-TGS devices demonstrate reduced responses to the combustion heat of H_2_ by integrating the Pt/*α*-Al_2_O_3_ catalyst at point B. Future work is planned to improve the combustion of CH_4_, for example, by changing the catalyst composition or materials.

## 4. Conclusions

Calorimetric-TGS devices were prepared by integrating catalyst combustors with a dual-catalyst structure (Pd/*θ*-Al_2_O_3_ and Pt/*α*-Al_2_O_3_ catalysts on the hot and cold sides, respectively). The calorimetric-TGS devices detected H_2_, CH_4_, and mixtures of the two with high resolution in the concentration range 200–2000 ppm (*i.e.*, the calorie range 0.51–15.9 J) and the temperature range 100–400 °C in air. Also, these calorimetric-TGS devices displayed both temperature- and gas concentration-dependent responses to H_2_, CH_4_, and the mixed gases with constant response-to-combustion heat conversion factors (*K*). The calorimetric-TGS device with the 3 wt% Pt/*α*-Al_2_O_3_ catalyst on the cold side reduced the response to H_2_ combustion heat by up to 50%. Therefore, we believe that this calorimetric-TGS device is a promising, low-cost, gas calorimeter candidate with short start-up and response times, and would be appropriate for small–systems applications. However, it will be necessary to achieve greater temperature accuracy and compensate for the dependence of the temperature measurement for the device in future work, because it is of crucial importance to measure the different gases in fuels with reliability and accuracy in calorimetric applications.

## Figures and Tables

**Table 1.** Calorimetric-TGS devices with various catalytic combustor compositions.

**Device**	**Catalyst**	
	**Point A**	**Point B**
Calorimetric-TGS #0	10 wt% Pd/*θ*-Al_2_O_3_	No catalyst
Calorimetric-TGS #1	10 wt% Pd/*θ*-Al_2_O_3_	0.3 wt% Pt/*α*-Al_2_O_3_
Calorimetric-TGS #2	10 wt% Pd/*θ*-Al_2_O_3_	3 wt% Pt/*α*-Al_2_O_3_
Calorimetric-TGS #3	10 wt% Pd/*θ*-Al_2_O_3_	30 wt% Pt/*α*-Al_2_O_3_

**Table 2.** Response-to-combustion heat conversion factors for the calorimetric-TGS devices.

**Device**	***K*** **(J/mV)**		
	*K*_*H*_2__	*K*_*CH*_4__	*K_Mixedgas_*
Calorimetric-TGS #1	1.6546	15.771	6.4081
Calorimetric-TGS #2	3.4524	17.874	9.3645
Calorimetric-TGS #3	2.0382	20.479	6.8084
